# Efficacy of Soft-Rot Disease Biocontrol Agents in the Inhibition of Production Field Pathogen Isolates

**DOI:** 10.3390/microorganisms11020372

**Published:** 2023-02-01

**Authors:** Jérémy Cigna, Kévin Robic, Pauline Dewaegeneire, Valérie Hélias, Amélie Beury, Denis Faure

**Affiliations:** 1 French Federation of Seed Potato Growers (FN3PT/inov3PT), 75008 Paris, France; 2 Institute for Integrative Biology of the Cell (I2BC), Paris-Saclay University, CEA, CNRS, 91190 Gif-sur-Yvette, France

**Keywords:** *Solanum tuberosum*, *Dickeya*, *Pectobacterium*, *gapA*, plant pathogen, pathogen population, field sampling, biocontrol

## Abstract

The *Dickeya* and *Pectobacterium* bacterial species cause blackleg and soft-rot diseases on potato plants and tubers. Prophylactic actions are essential to conserve a high quality of seed potato tubers. Biocontrol approaches are emerging, but we need to know how efficient biocontrol agents are when facing the natural diversity of pathogens. In this work, we sampled 16 production fields, which were excluded from the seed tuber certification scheme, as well as seven experimental parcels, which were planted with seed tubers from those production fields. We collected and characterized 669 *Dickeya* and *Pectobacterium* isolates, all characterized using nucleotide sequence of the *gapA* gene. This deep sampling effort highlighted eleven *Dickeya* and *Pectobacterium* species, including four dominant species namely *D. solani*, *D. dianthicola*, *P. atrosepticum* and *P. parmentieri*. Variations in the relative abundance of pathogens revealed different diversity patterns at a field or parcel level. The *Dickeya*-enriched patterns were maintained in parcels planted with rejected seed tubers, suggesting a vertical transmission of the pathogen consortium. Then, we retained 41 isolates representing the observed species diversity of pathogens and we tested each of them against six biocontrol agents. From this work, we confirmed the importance of prophylactic actions to discard contaminated seed tubers. We also identified a couple of biocontrol agents of the *Pseudomonas* genus that were efficient against a wide range of pathogen species.

## 1. Introduction

Several species of the genera *Dickeya* and *Pectobacterium* are causative agents of the blackleg and soft-rot diseases in *Solanum tuberosum* stems and tubers, respectively [1]. In Europe, the species currently isolated from blackleg and soft-rot lesions on potato plants encompass *Pectobacterium atrosepticum, Pectobacterium parmentieri, Pectobacterium brasiliense, Pectobacterium polaris, Dickeya dianthicola* and *Dickeya solani* [2]. *D. solani* emerged twenty years ago [3]. Some other species such as *P. punjabense*, *P. versatile* and *P. parvum* were less frequently detected in lesions of potato plants [4,5,6]. Over the past years, important efforts in genome sequencing of bacterial isolates deposited in collections and those collected by new samplings contributed to a more accurate delineation of *Dickeya* and *Pectobacterium* species (examples in [7,8,9,10]).

From this knowledge in taxonomy and genomics, different molecular tools have been developed for pathogen diagnosis, hence for identifying and discarding the contaminated plant materials from the production process of certified seed potato tubers [11]. For instance, in France, a quality scheme was set up along production of tuber seeds from the in vitro propagation of potato varieties until tuber harvest in the field. Before and after tuber harvest, intensive inspections are carried out in the frame of the official control and certification scheme implemented by SOC (French control and certification body). Before harvest, 1% of symptomatic plants is the highest threshold value of blackleg disease incidence for maintaining a given field in the production scheme. In post-harvest controls, the threshold value is set at 0.2% of dry and wet rots, expressed as a weight percentage of tubers (for details see http://frenchseedpotato.com, accessed on 20 November 2022). In France, each year between 2013 and 2017, which is the sampling period in this study, around 9000 lots were harvested from over 20,000 ha. Over this period, the mean rate of rejected plots was 0.85% per year. A maximum rate reached 2.1% in 2016 with particularly rainy conditions, which are more favorable to the development of the disease. The lowest rate was 0.3% in 2017, most likely because of drier environmental conditions (data from FN3PT http://frenchseedpotato.com, accessed on 20 November 2022).

In parallel to the pathogen diagnosis and prophylaxis approach, research has been carried out to identify potato varieties and genetic determinants involved in response to *Dickeya* and *Pectobacterium* pathogens [12,13]. Some others allowed the discovery of a wide variety of biological agents including phages and bacteria [14,15,16,17]. Because *Dickeya* and *Pectobacterium* pathogen populations are diverse and dynamic over time and space, we need to understand their structure in potato fields and evaluate the efficiency of the potential biocontrol agents using a refreshed collection of pathogen isolates.

In this work, we sampled 16 production fields that were rejected for producing seed potato tubers. We isolated 463 pathogens and compared the structure of the pathogen populations of these fields. In addition, we harvested asymptomatic tubers from thirteen of these rejected fields and we planted them the next year in experimental parcels. We isolated 206 additional pathogens and then we compared the structure of the pathogen populations in production fields (year N) and experimental parcels (year N + 1). Finally, 41 isolates that were representative of the collected species in the rejected potato fields were tested for their sensitivity against six biocontrol agents *Bacillus simplex* BA2H3, *Pseudomonas brassicacearum* PA1G7 and PP1-210F, *Pseudomonas fluorescens* PA3G8, *Pseudomonas lactis* PA4C2 and *Pseudomonas* sp. PA14H7 [14]. These biocontrol agents were previously identified after a screening of 10,000 bacterial isolates for their capacity to inhibit the growth of *P. atrosepticum* CFBP6276 and *D. dianthicola* RNS04.9 [14]. Hence, we could evaluate the biocontrol efficiency of these biocontrol agents against a wider range of pathogens that are representative of the species sampled in potato fields.

## 2. Materials and Methods

### 2.1. Isolation and Characterization of the Dickeya and Pectobacterium Pathogens

Between 2013 and 2017, 16 potato production fields (France) were sampled. These production fields were excluded from certified lots of seed tubers because they exhibited more than 1% of symptomatic plants (blackleg disease). In order to study *Pectobacterium* and *Dickeya* populations at the field scale, around 30 plants with blackleg symptoms were collected in each field, and a single bacterial isolate was retained from each of the 463 plants, resulting in 463 pathogen isolates.

Out of these 16 production fields, thirteen (P1 to P13) were used for plantation assays. From 800 to 4000 asymptomatic tubers were harvested (year N) in each field and then planted the next year (year N + 1) at the experimental station of Comité Nord (Achicourt, France). Out of the 13 experimental parcels, seven exhibited enough plants with blackleg symptoms for sampling: 206 diseased plants were collected (around 30 per parcel) and then 206 pathogens were isolated.

Our sampling approach revealed pathogen diversity at a field or parcel level but not at a plant individual level. In the case of production fields and experimental parcels, pectinolytic bacteria were isolated from lesions on crystal violet pectate medium [18]. Bacterial isolates were purified on agar plates and characterized at the genus (*Dickeya* and *Pectobacterium*) and species levels using the PCR primers listed in Table S1 [19,20,21,22,23,24]. Characterization of the *D. solani* and *D. dianthicola* isolates from the production fields P1 to P17 was already done in a previous paper [25].

### 2.2. Growth Inhibition Assays with Biocontrol Agents

Among the pathogen isolates sampled in potato fields between 2013 and 2017, 41 isolates representative of the species diversity were used in growth inhibition assays in triplicate with each of the six biocontrol agents: *Bacillus simplex* BA2H3, *Pseudomonas brassicacearum* PA1G7 and PP1-210F, *Pseudomonas fluorescens* PA3G8, *Pseudomonas lactis* PA4C2 and *Pseudomonas* sp. PA14H7 [14]. One hundred µL of each pathogen culture at 10^7^ CFU/mL were spread on a Petri dish in triplicate. After drying, 10 µL of each biocontrol agent culture at 10^9^ CFU/mL was spotted on the center of each Petri dish. After drying, the bacteria were incubated at 25 °C for 24 h. Then, the inhibition halo was measured for each pathogen strain—biocontrol strain combination.

### 2.3. Statistical Analyses

In the in vitro antibiosis tests, statistical analyses were performed using Rstudio (RStudio team, 2021.09.1 version, Boston, MA, USA, https://www.rstudio.com/, accessed on 20 November 2022) software for Windows using the packages FSA and agricolae. A Kruskal–Wallis test (alpha 0.05) followed by a Dunn test with Benjamini–Hochberg correction (alpha 0.05) was performed to compare each pathogen.

## 3. Results

### 3.1. Species Patterns in Rejected Productions of Seed Potato Tubers

**Figure 1:**
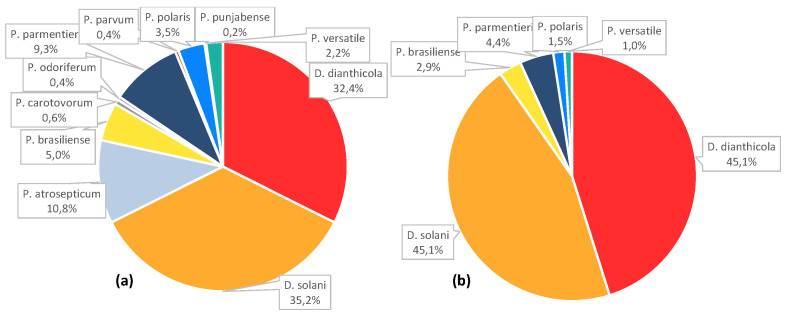
Species assignation. The *gapA* sequence was used for species assignation: (**a**) 463 isolates collected from 16 potato fields, which were rejected for seed tuber production because of blackleg symptoms, and (**b**) 206 isolates collected from seven parcels exhibiting blackleg symptoms after plantation of tuber seeds collected from symptomatic fields.

Sixteen potato fields, which were rejected for seed tuber production, were sampled in 2013 (fields P1 and P2), 2014 (P3, P4, P5 and P6), 2015 (P7, P8, P9 and P10), 2016 (P11, P12 and P13) and 2017 (P14, P15 and P17). Using a partial nucleotide sequence of *gapA*, the 463 pathogen isolates were assigned to eleven species. Only two Dickeya species, *D. dianthicola* (150 isolates) and *D. solani* (163), were identified. In contrast, a broad spectrum of Pectobacterium species was observed: *P. atrosepticum* (50 isolates), *P. brasiliense* (23), *P. carotovorum* (3), *P. odoriferum* (2), *P. parmentieri* (43), *P. parvum* (2), *P. polaris* (16), *P. punjabense* (1) and *P. versatile* (10). Among them, two species represented 62% of the *Pectobacterium* isolates (93 isolates out of 150): *P. atrosepticum* and *P. parmentieri*. *P. brasiliense*, *P. polaris* and *P. versatile* species were quite common (10 to 23 isolates) and the six other *Pectobacterium* species were rarely isolated (1 to 3 isolates) (Figure 1a).

Our sampling approach (one isolate from one symptomatic plant, repeated around 30 times in each experimental field) allowed us to access patterns of pathogen population in each field. Two to six species were identified in each field. Clearly, two patterns emerged among the analyzed fields, those enriched in *Pectobacterium* (P1, P3, P4 and P14) and the others in *Dickeya* species, either *D. solani* or *D. dianthicola*. Parcels P1, P3 and P4 contain only *Pectobacterium* isolates, while P14 contains also *Dickeya* isolates. In these four parcels enriched in *Pectobacterium* species, the relative abundance of the dominant species, namely *P. atrosepticum* in P1, P3 and P4 and *P. parmentieri* in P14, varies from 40% to 63%. In *Dickeya*-enriched patterns, *D. dianthicola* was dominant in P2, P7, P9, P12, P15 and P17 and *D. solani* in P5, P6, P8, P10, P11 and P13. Among the *Dickeya*-enriched patterns, the dominant species may represent up to 96% of the isolated strains.

In *Pectobacterium* species, the *gapA* nucleotide sequence revealed the presence of different alleles that give an insight into the infra-specific diversity of these pathogens. In the collected isolates, we identified two or three alleles in *P. atrosepticum*, *P. carotovorum*, *P. odoriferum* and *P. parmentieri*, and six in *P. brasiliense*, eight in *P. versatile* and 10 in *P. polaris*. Different alleles of the same species could coexist in the same production field (Table S2): for instance, three *gapA* alleles were identified in *P. parmentieri* isolates recovered from field P3, four *gapA* alleles in *P. brasiliense* from field P15 and five *gapA* alleles in *P. polaris* from field P1, suggesting the coexistence of different strains of the same species in the same field.

### 3.2. Species Patterns in Experimental Parcels

Out of the 16 production fields, thirteen (P1 to P13) were used for plantation assays using the harvested asymptomatic tubers. These production fields exhibited either a *Pectobacterium* pattern (P1, P3 and P4) or *Dickeya* pattern (P2, P5, P6, P7, P8, P9, P10, P11, P12 and P13). At the year N + 1, only seven of the planted experimental parcels (P2rep, P6rep, P7rep, P8rep, P9rep, P10rep and P12rep) exhibited enough plants with blackleg symptoms for sampling: 206 diseased plants were collected, and then 206 pathogens were isolated and characterized at the species level (Figure 1b).

**Figure 2:**
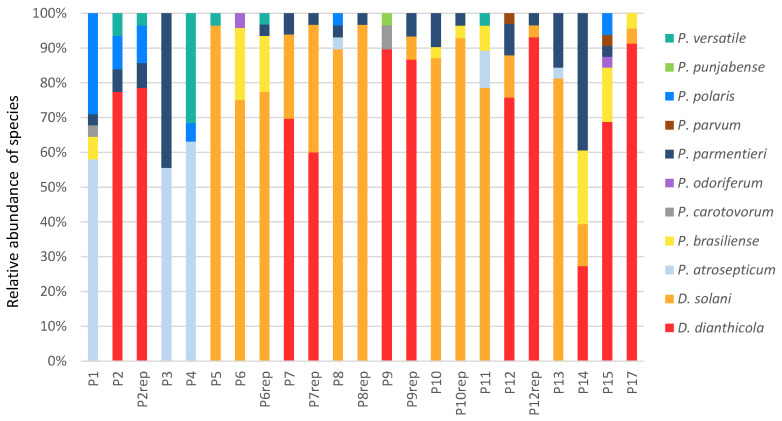
Species patterns in sampled fields and parcels. Relative abundance of species in sampled production fields (P1 to P17) and experimental parcels (P2rep to P12rep).

All seven sampled experimental parcels showed a *Dickeya* pattern (Figure 2). Seed tubers used for planting these experimental parcels were collected from production fields that also exhibited a *Dickeya* pattern, suggesting a vertical transmission of pathogens via tuber seeds. By comparing the species pattern (the relative abundance of species) of each pair of production fields and planted experimental parcel (P2 and P2rep, P6 and P6rep, P7 and P7rep, P8 and P8rep, P9 and P9rep, P10 and P10rep and P12 and P12rep), an exact Fischer test did not reveal a difference (all *p*-values > 0.3). Conservation of species pattern reinforced the hypothesis of vertical transmission of the pathogen consortia, including non-dominant species, in our experimental condition. At the subspecies level, a single *gapA* allele was identified in *D. solani* and *D. dianthicola* isolates, which dominate in experimental parcels.

### 3.3. Efficiency of Biocontrol Agents Facing Natural Diversity of Pathogens

We evaluated whether the biocontrol agents *B. simplex* BA2H3, *P. brassicacearum* PA1G7 and PP1-210F, *P. fluorescens* PA3G8, *P. lactis* PA4C2 and *Pseudomonas* sp. PA14H7 were active against the pathogens isolated from the sampled potato fields. We collected 738 measures of growth inhibition assays by testing in triplicate the six biocontrol agents against 41 pathogen isolates, all collected in production fields: five isolates of *D. dianthicola*, five of *D. solani*, seven of *P. atrosepticum*, five of *P. brasiliense*, five of *P. parmentieri*, four of *P. versatile*, three of *P. carotovorum*, three of *P. polaris*, two of *P. odoriferum*, one of *P. punjabense* and one of *P. parvum* (a list in Table S2).

**Figure 3:**
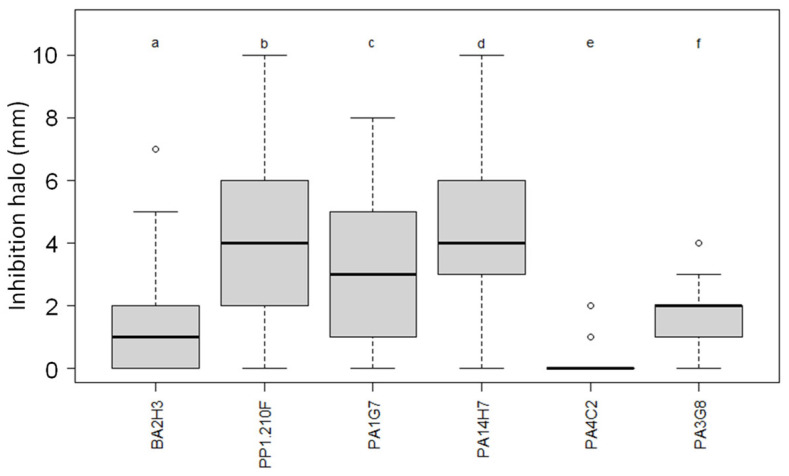
Growth inhibition assays of all six biocontrol agents. Growth inhibition assays were performed against 41 pathogen isolates in triplicate in the presence of each of the biocontrol agents: *B. simplex* BA2H3, *P. brassicacearum* PP1-210F and PA1G7, *Pseudomonas* sp. PA14H7, *P. fluorescens* PA3G8 and *P. lactis* PA4C2. In this graph, we calculated and showed mean value and variation of 123 halo values (41 pathogen isolates in triplicate) for each biocontrol agent. Statistical differences (Kruskal–Wallis test multiple comparison (Dunn), *p*-value adjusted with the Benjamini–Hochberg method, alpha = 0.05) are indicated by different letters (a to f); horizontal bar indicates mean value.

Considering all pathogen isolates, *Pseudomonas* sp. PA14H7 and *P. brassicacearum* PA1G7 and PP1-210F exhibited the highest growth inhibition capacity, while *P. fluorescens* PA3G8, *P. lactis* PA4C2 and *Bacillus simplex* BA2H3 were less efficient (Figure 3).

**Figure 4:**
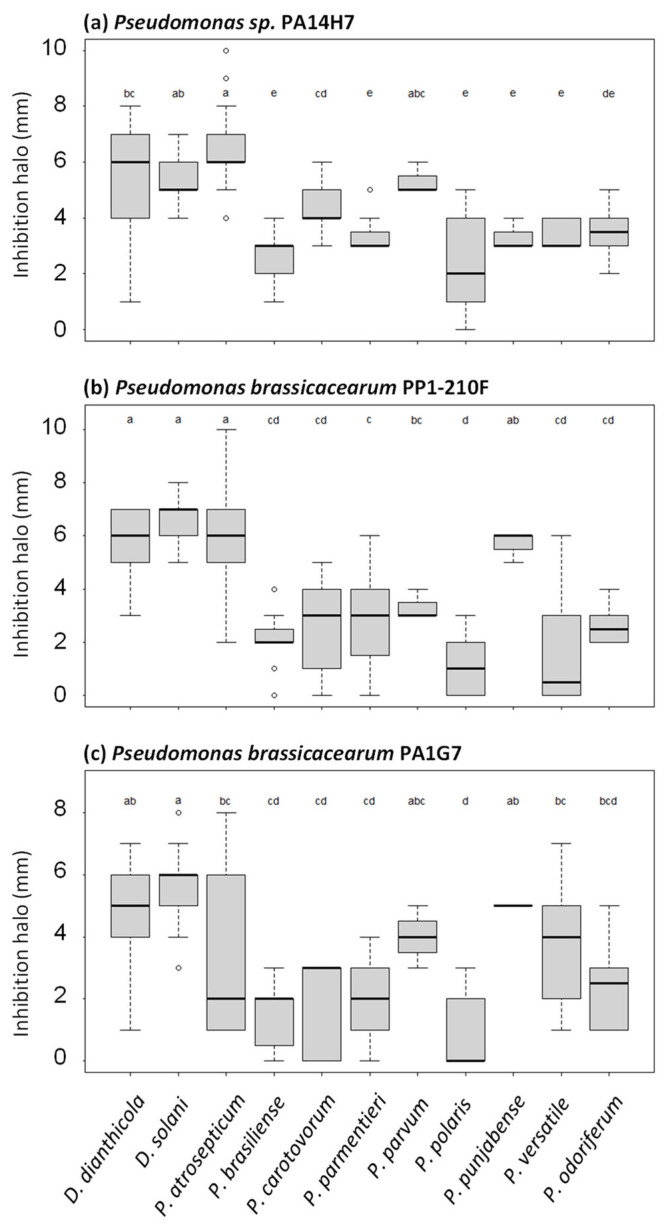
Refined analysis of growth inhibition halos caused by *Pseudomonas* sp. PA14H7 (**a**) and *P. brassicacearum* PP1-210F (**b**) and PA1G7 (**c**). The same data shown in Figure 3 were analyzed in a different way. For each biocontrol agent, we calculated mean value and variation for all halos collected with pathogen isolates belonging to the same species. Statistical differences (Kruskal–Wallis test multiple comparison (Dunn), *p*-value adjusted with the Benjamini–Hochberg method, alpha = 0.05) are indicated by different letters (a to e); horizontal bar indicates mean value.

**Figure 5:**
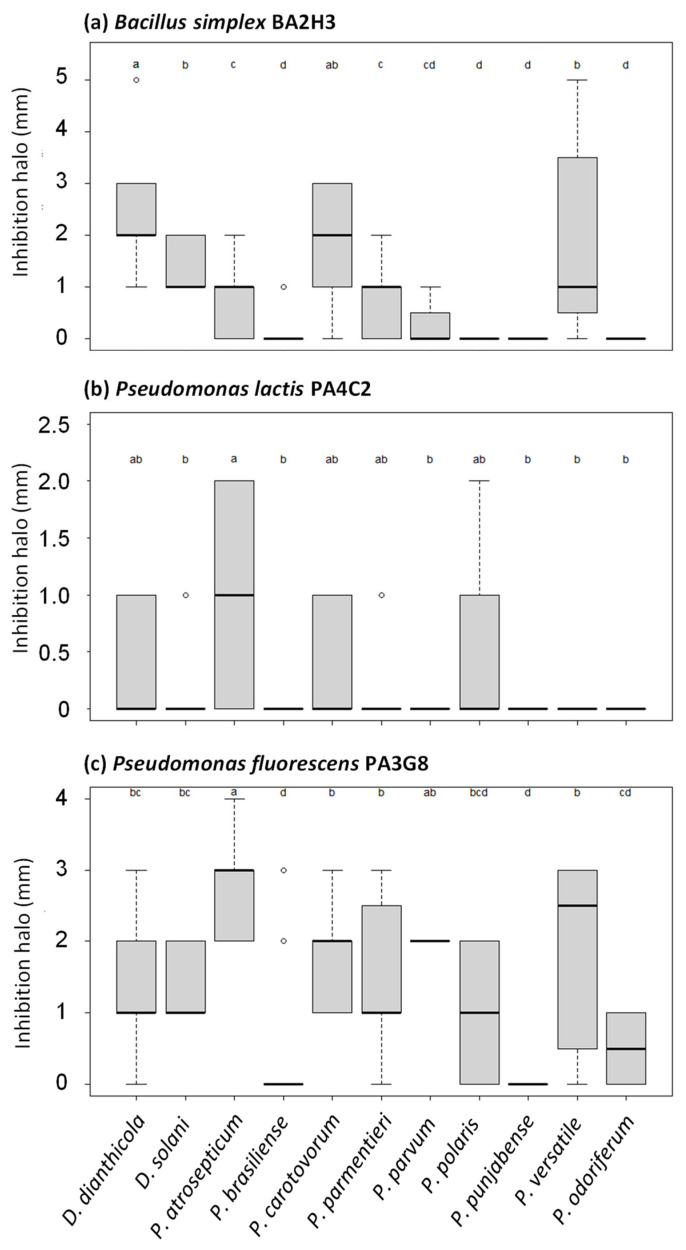
Refined analysis of growth inhibition halos caused by *B. simplex* BA2H3 (**a**), *P. lactis* PA4C2 (**b**) and *P. fluorescens* PA3G8 (**c**). The same data shown in Figure 3 were analyzed in a different way. For each biocontrol agent, we calculated mean value and variation for all halos collected with pathogen isolates belonging to the same species. Statistical differences (Kruskal–Wallis test multiple comparison (Dunn), *p*-value adjusted with the Benjamini–Hochberg method, alpha = 0.05) are indicated by different letters (a to d); horizontal bar indicates mean value.

In the next step, we refined our analysis of the collected 738 halo measurements by considering each pathogen species for each biocontrol agent (Figure 4 and Figure 5). *Pseudomonas* sp. PA14H7 and *P. brassicacearum* PA1G7 and PP1-210F were able to antagonize the growth of a broad spectrum of species (Figure 4). In the presence of *Pseudomonas* sp. PA14H7, the more sensitive pathogen species (classes a or b in statistical analysis) were *D. dianthicola, D. solani, P. atrosepticum* and *P. parvum*, while the other *Pectobacterium* species appeared as less sensitive (classes c, d or e in statistical analysis). In the presence of *P. brassicacearum* strains PA1G7 and PP1-210F, the more sensitive species were *D. dianthicola, D. solani, P. atrosepticum, P. parvum* and *P. punjabense,* whereas *P. versatile* also appeared as sensitive in the presence of *P. brassicacearum* strains PA1G7. Other results obtained with biocontrol strains *B. simplex* BA2H3, *P. lactis* PA4C2 and *P. fluorescens* PA3G8 are presented in Figure 5. These strains are less efficient for inhibiting the growth of the 41 tested pathogens.

## 4. Discussion

Several species-specific tools were developed for identifying and quantifying *Dickeya* and *Pectobacterium* pathogen species in plant samples, as well as for characterizing the collected bacterial isolates [11]. In this work, we mainly used a unique marker *gapA* for characterizing all the collected isolates from blackleg lesions. These symptomatic tissues were sampled in potato tuber production fields, which were excluded from tuber-seed production because of a high prevalence of blackleg symptoms. A strength of this PCR-sequencing tool is the absence of a priori on the taxon to be identified [24]. Another interest is the generation of nucleotide sequences that may be stored and used in alignments and relation trees with reference sequences according to an up-to-date taxonomy. For instance, this approach allowed us to identify a rare taxon *P. punjabense* in our collection of field isolates before the development of a dedicated species-specific tool [5]. The *gapA* marker is appropriate to uncover emerging or uncharacterized taxon.

In this work, we took advantage of the *gapA* marker for comparing *Dickeya* and *Pectobacterium* species patterns of potato tuber production fields. We focused our study on fields that were excluded from tuber-seed production because of a high prevalence of symptomatic plants (>1%). This work provided an understanding of what diversity of pathogens is discarded by the prophylactic strategy that is set up along the tuber-seed certification process. According to the relative abundance of the taxons in the 16 fields we sampled, we identified two major patterns: the *Pectobacterium* and *Dickeya* patterns. These patterns are characterized by a predominance of one species that represents at least 40% of the isolated pathogens. In this study, the predominant species were *D. solani*, *D. dianthicola*, *P. atrosepticum* and *P. parmentieri*. Together with predominant species, companion species were identified in each sampled field. Their numbers varied from one to five, among the following species: *D. solani*, *D. dianthicola*, *P. atrosepticum* and *P. parmentieri*, and among some less frequently isolated species such as *P. brasiliense, P. carotovorum, P. odoriferum, P. parvum, P. polaris, P. punjabense* and *P. versatile.* The predominance of some species could mirror a greater capability to exploit the potato host (hence a greater aggressiveness), and/or a greater competitiveness against other *Dickeya* and *Pectobacterium* pathogens and microbiota. Because only 16 fields were sampled, we could not exploit our data for testing statistical hypotheses of association or exclusion between species. We are currently expanding our samplings to investigate species relationships.

Several cases of predominant species and synergistic or antagonist relationships between *Dickeya* and *Pectobacterium* pathogens were reported. These relationships were considered at a species level, when behavior is shared by all (or almost all) strains of the same species, or at an infra-species level, when only one strain (or a few) exhibits a particular trait. In Norway, a study on 34 seed tuber lots revealed *P. atrosepticum* as a predominant species [26]. In Finland, a long-term survey (over 14 years) of seed tubers showed a predominance of *P. carotovorum* (52% of all samples) and, a more frequent co-occurrence of *Pectobacterium–Pectobacterium* species rather than *Pectobacterium–Dickeya* species [27]. In France, a long-term fields survey (over 10 years), revealed *Pectobacterium* species as predominant over *Dickeya* species in collected lesions and a non-random distribution of *Dickeya* and *Pectobacterium* predominant species in field survey [25]. In the USA, a field survey revealed *D. dianthicola* and *P. parmentieri* as predominant species; virulence assays in parcels showed *D. dianthicola* as more virulent than *P. parmentieri* [28]. A synergistic effect of the co-inoculation of the two species resulted in increased disease severity compared to single-species inoculation [28]. In Morocco, sampling revealed *P. brasiliense* and *D. dianthicola* as predominant species in distinctive production fields [29].

The predominant species reflects local contingencies that could be influenced by the impact of climatic factors: the optimal growth temperature is higher for *Dickeya* species as compared to *Pectobacterium* species, as discussed by Degefu et al. (2021) [27]. Relationships between *D. solani* and *D. dianthicola* appeared complex: competitive and synergistic behaviors were reported as depending on the colonized plant tissues. In greenhouse assays, *D. dianthicola* outcompeted *D. solani* in aerial parts of potato plants, while the two species co-existed in tubers [25]. In the case of *D. solani*, different properties were highlighted as potentially contributing to settlement in potato agrosystems: a capacity to initiate symptoms with a low inoculum in relation to a particular regulation of the *pelED* promoter [30]; a capacity to exploit a variety of nutrient resources [31], a capacity to produce a wide spectrum of anti-microbial compounds, improving its competitive fitness against other *Pectobacterium* and *Dickeya* species and microbiota [32,33]. Some other reports suggest that the production of antibiotics and their cognate resistance genes could also influence the dynamics of *Pectobacterium* and *Dickeya* pathogens by modifying the relative abundance of taxons and clones [34,35].

Facing the diversity of *Pectobacterium* and *Dickeya* pathogens, the main strategy deployed in all countries is a prophylactic approach consisting of discarding symptomatic parcels and lots of seed tubers. The identification and introgression of plant alleles for decreasing the sensitivity of *S. tuberosum* is a promising strategy, still in infancy [12,13]. Over the past years, intensive efforts allowed the identification of biocontrol agents: phages and bacteria [14,15,16,17]. A challenging issue is identifying biocontrol agents controlling a wide diversity of *Pectobacterium* and *Dickeya* species. Cocktails of phages or bacteria have been proposed to solve this issue [14,15,16], but an increased cost of production and patents could be expected. Another non-exclusive approach is to search for a biocontrol agent targeting a wide range of *Dickeya* and *Pectobacterium* species. In this work, we challenged six bacterial biocontrol agents against a wide diversity of pathogen isolates that we collected from potato fields. We showed that three of them exhibited a wide range spectrum in growth inhibition of pathogens. The biocontrol strain *Pseudomonas sp.* PA14H7 was very active, including against the predominant species that we identified in our sampling. The mechanism of action of biocontrol activity is under investigation.

## 5. Conclusions

This work highlighted distinctive patterns of *Dickeya* and *Pectobacterium* species in sampled production fields, which were excluded from the certification process of potato tuber seeds. Moreover, the *gapA* analysis revealed the infraspecific diversity at the plot scale and underlines the complex relationship existing between strains involved in the symptom expression. These patterns are maintained in parcels planted with rejected seed tubers, highlighting the importance of a certification scheme for rejecting contaminated tubers. Representatives of the *Dickeya* and *Pectobacterium* diversity in the potato fields were challenged by several biocontrol agents allowing the identification of a couple of biocontrol agents that were efficient against a wide range of pathogen species.

## Data Availability

All data are available in Table S1.

## Supplementary Materials

The following supporting information can be downloaded at: https://www.mdpi.com/article/10.3390/microorganisms11020372/s1, Table S1: Primers used in this study. Table S2: Characteristics of the collected *Dickeya* and *Pectobacterium* isolates.

## References

[1] Van Gijsegem F., Toth I.K., van der Wolf J.M., Van Gijsegem F., van der Wolf J.M., Toth I.K. (2021). Soft Rot Pectobacteriaceae: A Brief Overview. Plant Diseases Caused by Dickeya and Pectobacterium Species.

[2] Toth I.K., Barny M., Czajkowski R., Elphinstone J.G., Li X., Pédron J., Pirhonen M., Van Gijsegem F., Van Gijsegem F., van der Wolf J.M., Toth I.K. (2021). Pectobacterium and Dickeya: Taxonomy and Evolution. Plant Diseases Caused by Dickeya and Pectobacterium Species.

[3] van der Wolf J.M., Nijhuis E.H., Kowalewska M.J., Saddler G.S., Parkinson N., Elphinstone J.G., Pritchard L., Toth I.K., Lojkowska E., Potrykus M. (2014). *Dickeya solani* sp. nov., a Pectinolytic Plant-Pathogenic Bacterium Isolated from Potato (*Solanum tuberosum*). Int. J. Syst. Evol. Microbiol..

[4] Portier P., Pédron J., Taghouti G., Saux M.F.-L., Caullireau E., Bertrand C., Laurent A., Chawki K., Oulgazi S., Moumni M. (2019). Elevation of *Pectobacterium carotovorum* subsp. odoriferum to Species Level as *Pectobacterium odoriferum* sp. nov., Proposal of *Pectobacterium brasiliense* sp. nov. and Pectobacterium actinidiae sp. nov., Emended Description of Pectobacterium carotovorum and Description of *Pectobacterium versatile* sp. nov., isolated from Streams and Symptoms on Diverse Plants. Int. J. Syst. Evol. Microbiol..

[5] Cigna J., Laurent A., Waleron M., Waleron K., Dewaegeneire P., van der Wolf J., Andrivon D., Faure D., Hélias V. (2021). European Population of *Pectobacterium punjabense*: Genomic Diversity, Tuber Maceration Capacity and a Detection Tool for This Rarely Occurring Potato Pathogen. Microorganisms.

[6] Pasanen M., Waleron M., Schott T., Cleenwerck I., Misztak A., Waleron K., Pritchard L., Bakr R., Degefu Y., van der Wolf J. (2020). *Pectobacterium parvum* sp. nov., Having a Salmonella SPI-1-like Type III Secretion System and Low Virulence. Int. J. Syst. Evol. Microbiol..

[7] Khayi S., Cigna J., Chong T.M., Quêtu-Laurent A., Chan K.-G., Hélias V., Faure D. (2016). Transfer of the Potato Plant Isolates of *Pectobacterium wasabiae* to *Pectobacterium parmentieri* sp. nov. Int. J. Syst. Evol. Microbiol..

[8] Dees M.W., Lysøe E., Rossmann S., Perminow J., Brurberg M.B. (2017). *Pectobacterium polaris* sp. nov., Isolated from Potato (*Solanum tuberosum*). Int. J. Syst. Evol. Microbiol..

[9] Portier P., Pédron J., Taghouti G., Dutrieux C., Barny M.-A. (2020). Updated Taxonomy of *Pectobacterium* Genus in the CIRM-CFBP Bacterial Collection: When Newly Described Species Reveal “Old” Endemic Population. Microorganisms.

[10] Oulghazi S., Pedron J., Cigna J., Lau Y.Y., Moumni M., Gijsegem F.V., Chan K.G., Faure D. (2019). *Dickeya undicola* sp. nov., a Novel Species for Pectinolytic Isolates from Surface Waters in Europe and Asia. Int. J. Syst. Evol. Microbiol..

[11] Van der Wolf J.M., Cahill G., Van Gijsegem F., Helias V., Humphris S., Li X., Lojkowska E., Pritchard L., Van Gijsegem F., van der Wolf J.M., Toth I.K. (2021). Isolation, Detection and Characterization of Pectobacterium and Dickeya Species. Plant Diseases Caused by Dickeya and Pectobacterium Species.

[12] Joshi J.R., Brown K., Charkowski A.O., Heuberger A.L. (2022). Protease Inhibitors from *Solanum chacoense* Inhibit *Pectobacterium* Virulence by Reducing Bacterial Protease Activity and Mo-tility. MPMI.

[13] Lebecka R., Śliwka J., Grupa-Urbańska A., Szajko K., Marczewski W. (2021). QTLs for Potato Tuber Resistance to *Dickeya solani* Are Located on Chromosomes II and IV. Plant Pathol..

[14] des Essarts Y.R., Cigna J., Quêtu-Laurent A., Caron A., Munier E., Beury-Cirou A., Hélias V., Faure D. (2016). Biocontrol of the Potato Blackleg and Soft Rot Diseases Caused by *Dickeya dianthicola*. Appl. Environ. Microbiol..

[15] Carstens A.B., Djurhuus A.M., Kot W., Hansen L.H. (2019). A Novel Six-Phage Cocktail Reduces *Pectobacterium Atrosepticum* Soft Rot Infection in Potato Tubers under Simulated Storage Conditions. FEMS Microbiol. Lett..

[16] Maciag T., Krzyzanowska D.M., Jafra S., Siwinska J., Czajkowski R. (2020). The Great Five—An Artificial Bacterial Consortium with Antagonistic Activity towards *Pectobacterium* spp. and *Dickeya* spp.: Formulation, Shelf Life, and the Ability to Prevent Soft Rot of Potato in Storage. Appl. Microbiol. Biotechnol..

[17] Van der Wolf J.M., De Boer S.H., Czajkowski R., Cahill G., Van Gijsegem F., Davey T., Dupuis B., Ellicott J., Jafra S., Kooman M., Van Gijsegem F., van der Wolf J.M., Toth I.K. (2021). Management of Diseases Caused by *Pectobacterium* and *Dickeya* Species. Plant Diseases Caused by Dickeya and Pectobacterium Species.

[18] Hélias V., Hamon P., Huchet E., Wolf J.V.D., Andrivon D. (2012). Two New Effective Semiselective Crystal Violet Pectate Media for Isolation of *Pectobacterium* and *Dickeya*: Isolating Pectolytic Bacteria on CVP. Plant Pathol..

[19] Nassar A., Darrasse A., Lemattre M., Kotoujansky A., Dervin C., Vedel R., Bertheau Y. (1996). Characterization of *Erwinia chrysanthemi* by pectinolytic isozyme polymorphism and restriction fragment length polymorphism analysis of PCR- amplified fragments of *pel* genes. Appl. Environ. Microbiol..

[20] Darrasse A., Priou S., Kotoujansky A., Bertheau Y. (1994). PCR and restriction fragment length polymorphism of a pel gene as a tool to identify *Erwinia carotovora* in relation to potato diseases. Appl. Environ. Microbiol..

[21] Frechon D., Exbrayat P., Helias V., Hyman L.J., Jouan B., Llop P., Lopez M.M., Payet N., Perombelon M.C.M., Toth I.K. (1998). Evaluation of a PCR kit for the detection of *Erwinia carotovora* subsp. *atroseptica* on potato tubers. Potato Res..

[22] Kim M.H., Cho M.S., Kim B.K., Choi H.J., Hahn J.H., Kim C.K., Kang M.J. (2012). Quantitative real-time polymerase chain reaction assay for detection of *Pectobacterium wasabiae* using YD repeat protein gene-based primers. Plant Dis..

[23] Duarte V., de Boer S.H., Ward L.J., de Oliveira A.M. (2004). Characterization of atypical *Erwinia carotovora* strains causing blackleg of potato in Brazil. J. Appl. Microbiol..

[24] Cigna J., Dewaegeneire P., Beury A., Gobert V., Faure D. (2017). A *GapA* PCR-Sequencing Assay for Identifying the *Dickeya* and *Pectobacterium* Potato Pathogens. Plant Dis..

[25] Blin P., Robic K., Khayi S., Cigna J., Munier E., Dewaegeneire P., Laurent A., Jaszczyszyn Y., Hong K.W., Chan K.G. (2021). Pattern and Causes of the Establishment of the Invasive Bacterial Potato Pathogen *Dickeya solani* and of the Maintenance of the Resident Pathogen, *D. dianthicola*. Mol. Ecol..

[26] Rossmann S., Dees M.W., Torp T., Le V.H., Skogen M., Glorvigen B., van der Wolf J., Brurberg M.B. (2020). Field-Scale Molecular Testing of Virulent Potato Soft Rot Pectobacteriaceae in Norway. Eur. J. Plant Pathol..

[27] Degefu Y. (2021). Co-Occurrence of Latent Dickeya and Pectobacterium Species in Potato Seed Tuber Samples from Northern Finland. Agric. Food Sci..

[28] Ge T., Ekbataniamiri F., Johnson S.B., Larkin R.P., Hao J. (2021). Interaction between *Dickeya dianthicola* and *Pectobacterium parmentieri* in Potato Infection under Field Conditions. Microorganisms.

[29] Oulghazi S., Moumni M., Khayi S., Robic K., Sarfraz S., Lopez-Roques C., Vandecasteele C., Faure D. (2020). Diversity of Pectobacteriaceae Species in Potato Growing Regions in Northern Morocco. Microorganisms.

[30] Duprey A., Nasser W., Léonard S., Brochier-Armanet C., Reverchon S. (2016). Transcriptional Start Site Turnover in the Evolution of Bacterial Paralogous Genes—The *PelE-PelD* Virulence Genes in *Dickeya*. FEBS J..

[31] Raoul des Essarts Y., Pédron J., Blin P., Van Dijk E., Faure D., Van Gijsegem F. (2019). Common and Distinctive Adaptive Traits Expressed in *Dickeya Dianthicola* and *Dickeya Solani* Pathogens When Exploiting Potato Plant Host. Environ. Microbiol..

[32] Effantin G., Brual T., Rahbé Y., Hugouvieux-Cotte-Pattat N., Gueguen E. (2021). Dickeya Solani D S0432-1 Produces an Arsenal of Secondary Metabolites with Anti-Prokaryotic and Anti-Eukaryotic Activities against Bacteria, Yeasts, Fungi, and Aphids. bioRxiv.

[33] Matilla M.A., Monson R.E., Murphy A., Schicketanz M., Rawlinson A., Duncan C., Mata J., Leeper F., Salmond G.P.C. (2022). Solanimycin: Biosynthesis and Distribution of a New Antifungal Antibiotic Regulated by Two Quorum-Sensing Systems. mBio.

[34] Wang J.-W., Derilo R.C., Lagitnay R.B.J.S., Wu H.-P., Chen K.-I., Chuang D.-Y. (2020). Identification and Characterization of the Bacteriocin Carocin S3 from the Multiple Bacteriocin Producing Strain of *Pectobac-terium carotovorum* subsp. *carotovorum*. BMC Microbiol..

[35] Chung P.-C., Lagitnay R.B.J.S., Derilo R.C., Wu J.-L., Chuang Y., Lin J.-D., Chuang D.-Y. (2022). Unraveling the Uncharacterized Domain of Carocin S2: A Ribonuclease *Pectobacterium carotovorum* subsp. *carotovorum* Bacteriocin. Microorganisms.

